# Refining visual estimation of artificial chordae length using low fidelity simulator in non-resection technique of mitral valve repair

**DOI:** 10.1186/1749-8090-8-S1-P164

**Published:** 2013-09-11

**Authors:** A Hossien, I Khan, H Subhani, S Ashraf

**Affiliations:** 1 Cardiothoracic Surgery Department, Morriston Hospital, Swansea, UK

## Background

Non-resection technique is considered a valuable procedure in treatment of mitral valve (MV) incompetence using artificial chordae. The length of artificial chordae is a crucial step in the creation of optimal coaptation of leaflets. We propose low fidelity simulator to refine visual estimation of height of new chordae.

## Methods

The MV simulator was made from sponge with similar flexible properties to the normal MV components. PTFE chordae were used to create normal primary chordae during self-construction of the model. Different models with various pathologies were constructed. New Artificial chordae were placed in the pathological models utilizing diverse methods e.g. locked suture, multi-loop, chordae system technique, loop in loop and pre-measured chords. Chordal length was adjusted with visual estimation of leaflet coaptation to test optimal outcome in models. The tying ways of neochord on the leaflet edge are recorded e.g. atrial and ventricular site.

## Results

Self construction of normal 3D MV model results in understanding the geometry of leaflet coaptation, distribution of artificial chordae and variation of chordal length in relation to individual position. Locked suture technique was found to be simpler in achieving symmetrical coaptation in comparison with other chordae techniques which needed instrumental measurement in addition to visual estimation. Repetitive practising in pathological models refine visual estimation of adjusting chordae length, enhance the tying way to achieve optimal coaptation of leaflets and lead to reduced time spent in the operation room.

## Conclusion

Low fidelity simulation is a helpful and valuable tool in practising and refining artificial chordae length in mitral valve repair. Regular usage of the MV simulator results in minimizing learning curve and enhanced familiarity of performing repair with non-resection technique.

